# ZmRAP2.7, an AP2 Transcription Factor, Is Involved in Maize Brace Roots Development

**DOI:** 10.3389/fpls.2019.00820

**Published:** 2019-07-04

**Authors:** Jieping Li, Fanjun Chen, Yanqing Li, Pengcheng Li, Yuanqing Wang, Guohua Mi, Lixing Yuan

**Affiliations:** ^1^Key Laboratory of Plant-Soil Interaction, MOE, Department of Plant Nutrition, College Resources and Environmental Sciences, China Agricultural University, Beijing, China; ^2^Key Laboratory of Plant Stress Biology, State Key Laboratory of Cotton Biology, Department of Plant Science, School of Life Sciences, Henan University, Kaifeng, China; ^3^Jiangsu Key Laboratory of Crop Genetics and Physiology, Key Laboratory of Plant Functional Genomics, Co-Innovation Center for Modern Production Technology of Grain Crops, MOE, College of Agriculture, Yangzhou University, Yangzhou, China

**Keywords:** AP2 transcription factor, brace roots, candidate gene association, shoot-borne roots, root development, *Zea mays* L.

## Abstract

In maize, shoot-borne roots dominate the whole root system and play essential roles in water and nutrient acquisition and lodging tolerance. Shoot-borne roots initiate at shoot nodes, including crown roots from the belowground nodes and brace roots from aboveground nodes. In contrast to crown roots, few genes for brace roots development have been identified. Here, we characterized a maize AP2/ERF transcription factor, ZmRAP2.7, to be involved in brace roots development. *ZmRAP2.7* expressed in all types of roots, and the encoded protein localized in the nucleus with transcriptional activation activity. A maize transposon insert mutant *RAP2.7-Mu* defective in *ZmRAP2.7* expression revealed a decreased number of brace roots but not crown roots. Maize *Corngrass1* mutant, which showed an elevated expression of *ZmRAP2.7*, however, revealed an increased number of brace roots. The *ZmRAP2.7*-based association analysis in a maize panel further identified a SNP marker at the fifth exon of gene to be associated with number of brace roots. These results uncovered a function of ZmRAP2.7 in brace roots development and provided the valuable gene and allele for genetic improvement of maize root systems.

## Introduction

Roots are essential organs for exploring and exploiting soil resources, such as water and mineral nutrients, and providing anchorage (Lynch, 1995, 2007; Hodge et al., 2009). Root system architecture (RSA) is fundamental for crop growth and productivity, particularly under abiotic stress condition (de Dorlodot et al., 2007). Maize (*Zea mays* L.) is one of the most important food and feed crop, and the productivity is sensitive to nutrient deficiency, drought stress, and root lodging in high-density populations (Mueller et al., 2012; Lobell et al., 2014; Xue et al., 2017). A simulation study in the USA Corn Belt suggested that the historical maize yield trend can be explained by the improvement of root systems (Hammer et al., 2009). The deep root system is proposed to be optimal for water and nitrogen acquisition, while the shallow root system is suitable for phosphorus absorption (Lynch, 2011, 2013). However, since it is difficult to evaluate roots traits under field conditions, plant breeders are seldom considered root traits as a selection criterion (Cai et al., 2012). Therefore, identification of QTLs or genes for RSA is required to provide the valuable targets for marker-assisted selection or genetic modification to promote root-based approach on crop yield improvement.

In maize, the root systems are formed during the embryogenesis and post-embryonic development (Hochholdinger and Tuberosa, 2009). The post-embryonic shoot-borne roots dominate the whole root system, representing the major components for resource acquisition and lodging tolerance for the adult plants. Shoot-borne roots are initiated at the consecutive shoot nodes and consist of crown roots (CR) from belowground nodes and brace roots (BR) from aboveground nodes (Hochholdinger, 2009). Using maize *rootless concerning crown and seminal roots* (*rtcs*) mutant, *ZmRTCS* gene have been identified for the involvement in developing shoot-borne roots (Taramino et al., 2007). *ZmRTCS* and its close homolog gene *ZmRTCL* both encoded LOB domain proteins that regulated shoot-borne roots initiation and elongation, respectively (Taramino et al., 2007; Xu et al., 2015). Both genes act as the downstream of auxin response factor *ZmARF35*, suggesting a role of auxin pathway in shoot-borne roots development (Xu et al., 2015). Using a teosinte-maize population, a flowering-time gene *ZmCCT* was also found to control number of shoot-borne roots (Zhang et al., 2018). For adult maize plants, the developed brace roots grown into the soil are mainly responsible for nutrients and water absorption and against lodging. The anatomical, morphological, and transcriptomic pattern of brace roots significantly differed from those of crown roots, suggesting the genetic differences between these two types of roots in maize (Li et al., 2011; Yu et al., 2015, 2016). Some QTLs for brace roots traits have been mapped (Cai et al., 2012; Ku et al., 2012; Gu et al., 2016), but the underlying genes have not yet been identified. A multidrug-and-toxin-extrusion (MATE) transporter gene may confer to brace roots development as the corresponding mutant *big embryo1* revealed more number of brace roots than the wild-type plants (Suzuki et al., 2015). Nevertheless, the molecular mechanism of brace roots development is still poorly understood.

Some AP2 transcription factors were found to be involved in regulating crown roots development in rice (Zhao et al., 2009; Kitomi et al., 2011). For example, *CROWNROOTLESS5* (*CRL*5) is involved in crown roots initiation (Kitomi et al., 2011), and another AP2 transcription factor, *ERF3* can interact with *WOX11* to control crown roots initiation and development (Zhao et al., 2009, 2015). The AP2 transcription factors belong to a large gene family of plant transcription factors, which contain the highly conserved AP2/ERF DNA-binding domain. The AP2 members were assigned into four subfamilies: AP2, RAV (related to ABI3/VP1), dehydration-responsive element-binding protein (DREB), and ERF (Yamaguchi-Shinozaki and Shinozaki, 2006; Dietz et al., 2010; Sharoni et al., 2011; Mizoi et al., 2012). Phylogenetic analysis predicted a total of 184 AP2-like genes in maize genome (Du et al., 2014), and none of them was reported to regulate root development.

Maize *Corngrass1* mutant with elevated expression of *miR156* exhibited more brace roots (Chuck et al., 2007a). As the downstream of *miR156, miR172* was depressed in *Corngrass1* (Chuck et al., 2007b). Since some member of AP2/ERF (AP2) transcription factors act as the targets of *miR172*, the miR156-miR172-AP2 pathway was proposed to be a regulatory pathway that controlled the timing of the juvenile-to-adult phase transition and subsequently influenced flowering time and floral development (Irish, 1997; Chuck et al., 2007a,b; Buckler et al., 2009; Castelletti et al., 2014). As the elevated expression of an *AP2* gene, *ZmRAP2.7*, as the target of *miR172*, was observed in the node with brace roots of *Corngrass1*, we then suspected the role of *ZmRAP2.7* in maize brace roots development. The *ZmRAP2.7* encoded protein was localized in the nucleus with the activity of transcriptional activation. The maize mutant *RAP2.7-Mu* defective in *ZmRAP2.7* expression revealed a decreased number of brace roots. Thus, this finding suggested the function of *ZmRAP2.7* in maize brace roots development.

## Materials and Methods

### Plant Stocks and Growth Conditions

The *ZmRAP2.7* Mu-transposon insertion mutant (Stock ID: UFMu-00629; Locus ID: mu1019979), named *RAP2.7-Mu*, was obtained from the Uniform-Mu project in the Maize Genetics Cooperation Stock Center^1^ (McCarty et al., 2005). The corresponding wild-type inbred line W22 was grown in a climate-controlled greenhouse (16/8 h light-dark cycle at temperatures of 29/24°C). For RNA extraction, different organs were collected at the silking stage, including root, the node with or without brace roots. The *Corngrass1* mutant was deposited in the Maize Genetics Cooperation Stock Center as *CG1* (Stock ID: 310D; Chuck et al., 2007a).

The *RAP2.7-Mu* and W22 plants were grown in the field at Shangzhuang (SZ) Experimental Station (Beijing, N40°08′12.15″, E116°10′44.83″) during the summers in 2016 and 2017 and at Sanya (SY) Experiment Station (Hainan, N18°22′55.83″, E109°11′43.94″) during the winter in 2016. Leaf samples were collected at the seedling stage, and the genomic DNA was then extracted for the genotyping. A BCF_1_ segregation population from the cross of *Corngrass1* and W22 line was planted, and the node tissue with the primordia of brace roots was then sampled for RNA extraction.

### Cloning and Sequence Analysis of *ZmRAP2.7*

The reference sequence of *ZmRAP2.7* (*GRMZM2G700665*) was identified from Phytozome database^2^. Full length of *ZmRAP2.7* cDNA was amplified using the specific primers located at 5′-UTR and 3′-UTR regions (Supplementary Table S1). The PCR product was then ligated into CloneSmarter-TOPO vector (TaiHe Biotechnology, Beijing, China) and sequenced. The structure of *ZmRAP2.7* encoded protein was predicted by SMART^3^. The secondary structure was predicted by PSIPRED^4^ and the tertiary structure by SWISS-MODEL^5^.

### Subcellular Localization of ZmRAP2.7 Protein

The coding sequence of *ZmRAP2.7* was cloned into the BamHI and EcoRI sites of the *pEZS-NL* transient expression vector under the control of the *35S* promoter to generate *pEZS-35S:ZmRAP2.7-EGFP* construct (Zuo et al., 2015). Maize protoplasts were isolated from etiolated maize seedlings of inbred B73 for transformation as described by Yoo et al. (2007). After the incubation at 24°C for 12 h in the dark, GFP fluorescence in the transformed protoplasts was visualized using a LSM510 META confocal scanning laser inverted microscope (Carl Zeiss, Jena, Germany).

### Transactivation Activity Assay of ZmRAP2.7 Protein

*ZmRAP2.7* cDNA was cloned into the EcoRI and BamHI sites of *pGBKT7* vector to generate *pGBKT7-ZmRAP2.7* construct. This plasmid with empty vector control was then transformed into yeast strain AH109 to analysis the transactivation activity. Yeast transformants with OD600 of 0.1 were plated on various selective media, SD/-Trp and SD/-Trp-His, and incubated at 30°C for 3 days.

### Phylogenetic Analysis of ZmRAP2.7

To analysis phylogenetic tree of ZmRAP2.7, the peptide sequences of selected members of AP2 transcription factor subfamily of *Oryza sativa, Sorghum bicolor, Populus trichocarpa*, and *Arabidopsis thaliana* were obtained from the database. The peptide sequences of 28 maize AP2 members were obtained from PlantTFDB database^6^. The sequence alignment was performed by the software DNAMAN, MEGA (version 6) (Tamura et al., 2007) and ClustalX2.0 (Larkin et al., 2007). Phylogenetic tree was constructed using the neighbor-joining method (Saitou and Nei, 1987).

### Gene Expression Analysis

Total RNA was isolated using Trizol reagent (Takara, Dalian, China) and treated with DNaseI to eliminate genomic DNA contamination. The cDNA was amplified using a PrimeScript™ RT reagent Kit with gDNA Eraser kits (Takara, Dalian, China). Expression of *ZmRAP2.7* was analyzed by qRT-PCR method (Bio-Rad, Hercules, CA, United States) using the fluorescent DNA intercalating dye SYBR Green I Master Mix (TAKARA, Dalian, China). The gene-specific oligonucleotide primers for the gene expression analysis were designed, and the efficiency and specificity of the candidate primers were examined by a melting curve analysis from 55 to 99°C. All primer sequences are listed in Supplementary Table S1. Three biological replicates and three technical repetition were performed. The thermal cycling program was as follows: 40 cycles at 95°C for 3 min, 95°C for 10 s, and 58°C for 30 s. Expression levels were normalized to the maize *ubiquilin-1* (*ZmUBQ1*) gene as an internal control, and the data were analyzed based on the comparative 2-ΔΔCT formula (Livak and Schmittgen, 2001).

### Identification of Mu Transposon Insertion Line *RAP2.7-Mu*

The maize genotype was identified by the method as described by Settles et al. (2007). Two gene-specific primers AP2SF and AP2SR were designed beside the predicted insertion position sequence in the chromosome 8 (131,577,941~131,577,949) (B73 RefGen_v3). Combined with a Mu-TIR-specific primer TIR6 (McCarty et al., 2005), the transposon fragments were amplified and the corresponding insertion site was verified. Plant genotypes were analyzed using primers sets AP2SF + AP2SR and AP2SR + TIR6, and the homozygous for either wild type or mutant and the heterozygote were then identified (Supplementary Figure S1).

### Root Phenotyping

The number of shoot-borne roots, including brace roots and crown roots, was evaluated for the different maize genotypes (*RAP2.7-Mu, Corngrass1*, and the corresponding wild types) by a modified “shovelomics” method (Trachsel et al., 2011). Roots of field-grown maize plants were excavated and then cleaned by removing the soil. Each brace roots and crown roots were cut following the order of node, and the number of roots was counted. The order of whorl was recorded from the bottom to upper nodes (Supplementary Figure S2). Significant difference between different genotypes was determined by Student’s *t*-tests (^*^p ≤ 0.05; ^**^p ≤ 0.01; ^***^p ≤ 0.001).

### Candidate Gene Association Analysis of *ZmRAP2.7* With Root Traits in the AM508 Panel

A maize association panel composed of 508 diverse inbred lines (AM508) was used to analyze the traits of number of brace and crown roots under the field conditions (Li et al., 2013). Root phenotype of each inbred lines was evaluated at Shangzhuang (SZ) Experimental Station (Beijing, N40°08′12.15″, E116°10′44.83″) and Quzhou (QZ) Experimental Station (Hebei, N36°51′48.55″, E115°00′58.62″) during the summer in 2012. At each location, all lines were planted in one-row plots with an incompletely random design. Each row was 4 m long, 0.5 m wide, and contained 17 plants. Root traits of five randomly selected plants per row were evaluated. The best linear unbiased predictor (BLUP) values from two locations were used for the association analysis. Polymorphisms (single-nucleotide polymorphism, SNPs) of *ZmRAP2.7* were obtained from database^7^, and their association to the investigated traits was calculated by TASSEL5.0, under the standard MLM, with MAF ≥ 0.05.

## Results

### Elevated Expression of *ZmRAP2.7* in *Corngrass1* Revealed More Brace Roots

**Figure 1:**
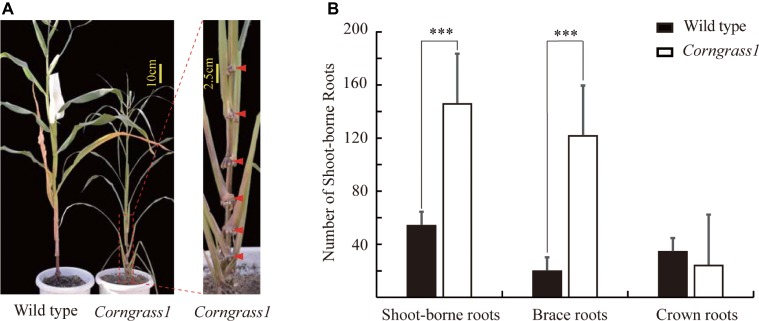
Increased number of brace roots in *Corngrass1* mutant. **(A)** *Corngrass1* mutant and wild-type plants were grown in greenhouse for 68 days. Upper nodes in *Corngrass1* still maintained at juvenile phase and initiated shoot-borne roots. **(B)** Number of shoot-borne roots, which consist of brace and crown roots, in field-grown *Corngrass1* mutant and wild-type plants at mature stage. Bars indicate mean (±SD) (*n* = 6). Significant difference within each group was indicated by an asterisk (^***^p < 0.001) according to Student’s *t*-tests.

The maize *Corngrass1* mutant developed more brace roots than its corresponding wild type (Figure 1A). In the field, the number of shoot-borne roots averaged 122.3 in *Corngrass1*, about six fold more than that wild-type plants (Figure 1B). Increased number of shoot-borne roots in *Corngrass1* was mainly explained by brace roots, while the number of crown roots was similar between two genotypes. The previous study showed that in *Corngrass1* expression of *miR156* was elevated and subsequently repressed *miR172* (Navarro et al., 2017). *ZmRAP2.7*, one of the putative target genes of *miR172*, was then supposed to be upregulated in *Corngrass1* and probably responded to the brace roots phenotype.

**Figure 2:**
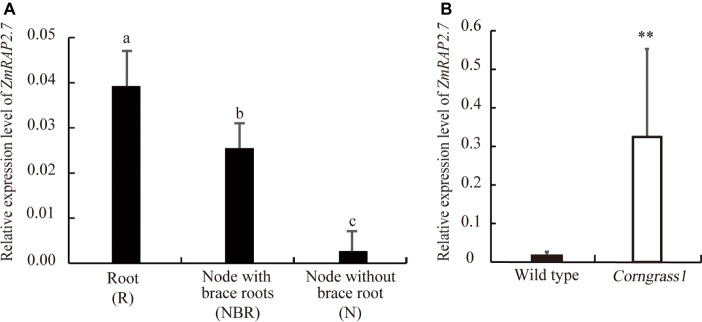
Preferential expression of *ZmRAP2.7* in nodes initiated brace roots and upregulation in *Corngrass1* mutant. **(A)** Relative expression levels of *ZmRAP2.7* in root (R), nodes with brace roots (NBR), and nodes without brace roots (N) in field-grown wild-type plants at flowering stage. Bars indicate mean (±SD) (*n* = 3). Significant difference was indicated by different letters according to Tukey’s test. **(B)** Relative expression levels of *ZmRAP2.7* in nodes of field-grown *Corngrass1* mutant and wild-type plants at flowering stage. Bars indicate mean (±SD) (*n* = 3). Significant difference was indicated by an asterisk (^**^p < 0.01) according to Student’s *t*-tests.

To define the function of *ZmRAP2.7* in maize brace roots development, the expression pattern of *ZmRAP2.7* in different organs was surveyed from database (B73 genome V3, https://www.maizegdb.org/). *ZmRAP2.7* expression levels were abundant in all types of roots, including primary, seminal, and shoot-borne roots (Supplementary Figure S3). We further examined *ZmRAP2.7* expression in root (R), node with brace roots (NBR), and node without brace roots (N) of plants at the silking stage (Supplementary Figure S4). Again, *ZmRAP2.7* showed the highest expression levels in roots. Remarkably, *ZmRAP2.7* expression in the node with the primordia of brace roots was about 10 times higher than that in the node without the primordia of brace roots (Figure 2A), suggesting that *ZmRAP2.7* may function in brace roots development. Furthermore, in the developed nodes at the silking stage, *ZmRAP2.7* expression in *Corngrass1* was about 30-fold higher than that in wild type (Figure 2B). Thus, the elevated expression of *ZmRAP2.7* in the node may explain the more number of brace roots in *Corngrass1*.

**Figure 3:**
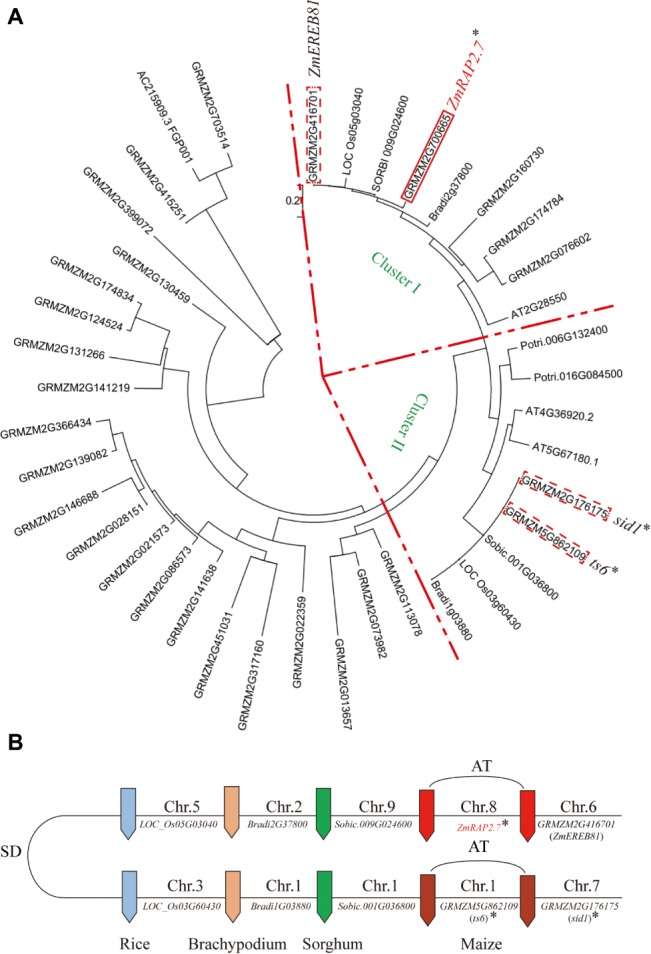
Phylogenetic tree and comparative genome analysis of *ZmRAP2.7* and its homologs in different grass species. **(A)** The phylogenetic tree was constructed using MEGA software. *GRMZM2M2G700665* (*ZmRAP2.7*), *Sobic.009G024600, GRMZM2G416701* (*ZmEREB81*), *Os05G03040,* and *Bradi2G37800* were assigned into Cluster I. *GRMZM5G862109* (*ts6*), *GRMZM5G176175* (*sid1*), *Sobic.001G036800, Os03G60430*, and *Bradi1G03880* were assigned into Cluster II. **(B)** Schematic representation of the chromosome locations of *ZmRAP2.7* and homolog genes within the framework of *Poaceae* inter- and intra-species collinearity. The clusters I and II resulted from duplicated segments of the *Poaceae* genome originating from the ancestral whole genome duplication (SD) and a pair of two genes within each cluster from the genome allotetraploidization (AT). Genes contained the targeted sites of *miR172* were indicated with an asterisk.

By the phylogenetic analysis, *ZmRAP2.7* (*GRMZM2M2G700665*) and the close homolog *ZmEREB81* (*GRMZM2G416701*) were assigned into the Cluster I and *ts6* (*tassel seed6, GRMZM5G862109*) and *sid1* (*sister of indeterminate spikelet 1, GRMZM5G176175*) into the Cluster II (Figure 3A). The clusters I and II were probably caused by duplicated segments of the *Poaceae* genome originating from the ancestral whole genome duplication (Figure 3B). A pair of two genes in each cluster was resulted from the genome allotetraploidization. In the Cluster II, both *ts6* and *sid1* were the target genes of *miR172* and function in floral organ identity (http://www.mirbase.org/; Chuck et al., 2007b). The *ts6* and *sid1* were constitutively expressed in most of organs as revealed by database (http://www.maizegdb.org/; Stelpflug et al., 2016) (Supplementary Figure S3). In the Cluster I, both *ZmRAP2.7* and *ZmEREB81* were highly expressed in roots. Since *ZmEREB81* lost the *miR172* target site, *ZmRAP2.7* was supposed to be the candidate gene for regulating root development.

### Number of Brace Roots Decreased in *ZmRAP2.7* Transposon Insertion Mutant

**Figure 4:**
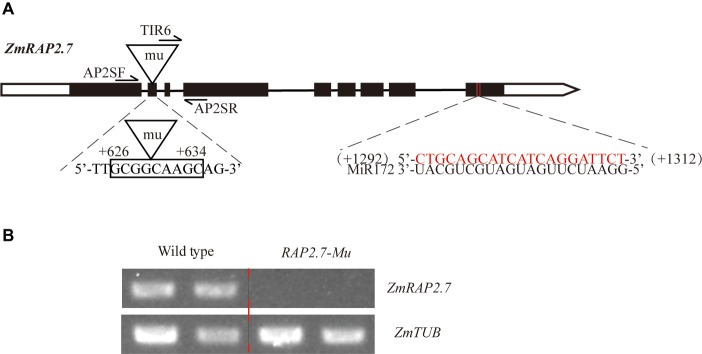
Isolation of *RAP2.7-Mu* transposon insertion mutant defective in *ZmRAP2.7* expression. **(A)** A *Mu* transposon was inserted in the second exon of *ZmRAP2.7* gene at the position between +626 and +634 bp with the two 9 bp repeats 5′-GCGGCAAGC-3′. The *miR172* target sequence was located in the ninth exon at the position between +1,292 and +1,312 bp. Genotypes were analyzed using primers sets AP2SF + AP2SR and AP2SR + TIR6. **(B)** Expression levels of *ZmRAP2.7* in roots of the *RAP2.7-Mu* and the corresponding wild-type plants grown in pots.

To verify the role of *ZmRAP2.7* in brace roots development, we characterized the Mu-transposon insertion mutant (*RAP2.7-Mu*) in which *ZmRAP2.7* expression was disrupted. By sequencing the PCR products amplified from Mu-specific and *ZmRAP2.7* gene-specific primers, the Mu transposon was found to be inserted into the second exon of *ZmRAP2.7* gene at +626 to +634 bp region with a repeat sequence 5′-GCGGCAAGC-3′ (Figure 4A). By the semi-quantitative RT-PCR analysis of seedlings root sample, *ZmRAP2.7* expression in wild-type plants was revealed as an apparent band while no signal was detected in *RAP2.7-Mu* (Figure 4B). Thus, this result indicated that *ZmRAP2.7* expression was completely disrupted in *RAP2.7-Mu* by a transposon insertion into the coding sequence.

**Figure 5:**
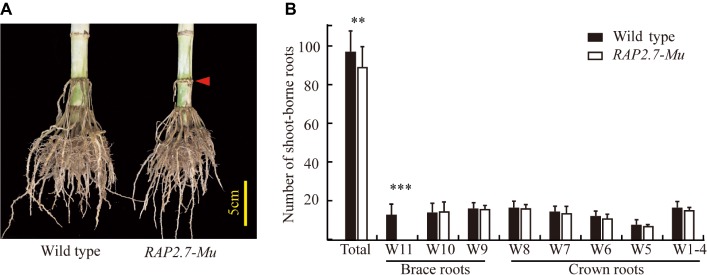
Decreased number of brace roots in *RAP2.7-Mu* mutant as revealed by F_2_ segregating population. **(A)** Phenotype of brace roots development in *RAP2.7-Mu* mutant and wild-type W22 plants grown in the field at mature stage (75 days after sowing). The difference in brace roots between two genotypes was signed with a red arrow. **(B)** Number of brace and crown roots of maize plants grown in Shangzhuang (2016) at mature stage (75 days after sowing). The *RAP2.7-Mu* mutant and wild-type W22 plants were genotypically selected from a F_2_ segregating population. The whorl corresponding to node numbers in orders was indicated as W1–W11. Bars indicate mean (±SD) (*n* = 23–28). Significant difference within each group was indicated with an asterisk (^**^p < 0.01; ^***^p < 0.001) according to Student’s *t*-tests.

The number of shoot-borne roots, including brace and crown roots, was then compared between *RAP2.7-Mu* and the corresponding wild-type plants under the field conditions. To rule out the possible influence of other insertions or genomic modifications on the investigated traits in *RAP2.7-Mu*, we generated a F_2_ segregating populations from the cross of *ZmRAP2.7-Mu* and wild-type W22, and the association between *ZmRAP2.7-Mu* allele and root phenotype was then analyzed. In the field trials at SZ2016, by genotyping of each individual within the F_2_ population, we obtained 28 plants with homozygous *ZmRAP2.7-Mu* allele (−/−), 60 plants with heterozygous *ZmRAP2.7-Mu* allele (−/+), and 23 plants with homozygous wild-type allele (+/+), presenting a segregation ratio of 1:2:1. On average, the homozygous *RAP2.7-Mu* plants had significantly 8.6% less number of shoot-borne roots (89.4) than those with wild-type allele (97.8) (Figures 5A,B), while no difference was observed between heterozygous and wild-type allele. Thus, this indicated that the decrease number of brace roots in *ZmRAP2.7-Mu* resulted from a recessive mutation in *ZmRAP2.7*. Number of crown roots in each whorl (W1–W8) was similar between both genotypes. By contrast, less brace roots observed in *RAP2.7-Mu* plants were mainly explained by one whorl less (W9, W10) than that of wild type (W9–W11). Therefore, the disruption of *ZmRAP2.7* expression significantly inhibited the development of brace roots rather than crown roots. In addition, the homozygous *RAP2.7-Mu* also showed an early pollen shed time than the homozygous wild type (8 days earlier in SZ2016 and 4 days in SZ2017) (Supplementary Table S3). Besides brace roots development, the role of *ZmRAP2.7* in flowering time was also confirmed as described by Salvi et al. (2007).

**Figure 6:**
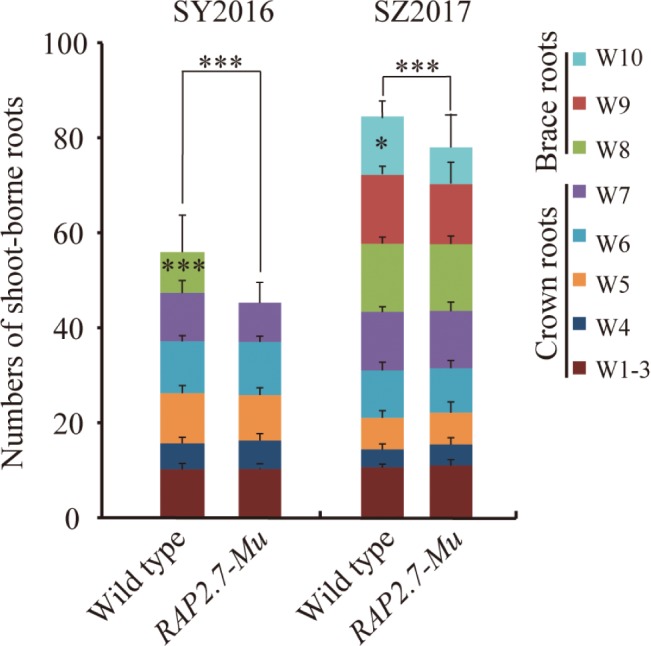
Decreased number of brace roots in *RAP2.7-Mu* mutant as revealed by F_2:3_ families. Number of brace and crown roots of maize plants grown in Sanya (SY2016) and Shangzhuang (SZ2017) at mature stage (about 78 days after sowing). The F_2:3_ families’ plants of *RAP2.7-Mu* mutant and wild-type W22 were compared. The whorl corresponding to node numbers in orders was indicated as W1–W10. Bars indicate mean (±SD) (*n* = 36). Significant difference within each group was indicated by an asterisk (^***^p < 0.001) according to Student’s *t*-tests.

To confirm the root phenotype of *RAP2.7-Mu*, we further generated the F_2:3_ families from either homozygous *RAP2.7-Mu* or homozygous wild-type plants. The root phenotypes were evaluated in SY2016 and SZ2017. In SY2016, the *RAP2.7-Mu* showed about 42.0 shoot-borne roots on average, about 14% significant reduction compared with wild type (Figure 6). The wild-type plants developed the brace roots in the eighth whorl (W8), while *RAP2.7-Mu* failed to initiate the brace roots at the same whorl (Figure 6). The number of crown roots (W1–W7) was similar between both genotypes. Compared to plants in SY2016, number of shoot-borne roots, mainly for brace roots, significantly increased in SZ2017, indicating the brace roots development also influenced by the environments. Nevertheless, number of shoot-borne roots in *RAP2.7-Mu* (72.5) also decreased up to 12% than that in wild type (82.7) (Figure 6). Again, the decrease was mainly explained by the less number of brace roots in the 10th whorl (W10). In addition, the root dry weight showed about 70% reduction in *RAP2.7-Mu* plants, while the shoot dry weight remained similar between two genotypes (Supplementary Figure S5). Collectively, these results indicated that *ZmRAP2.7* was involved in the development of brace roots rather than crown roots.

**Figure 7:**
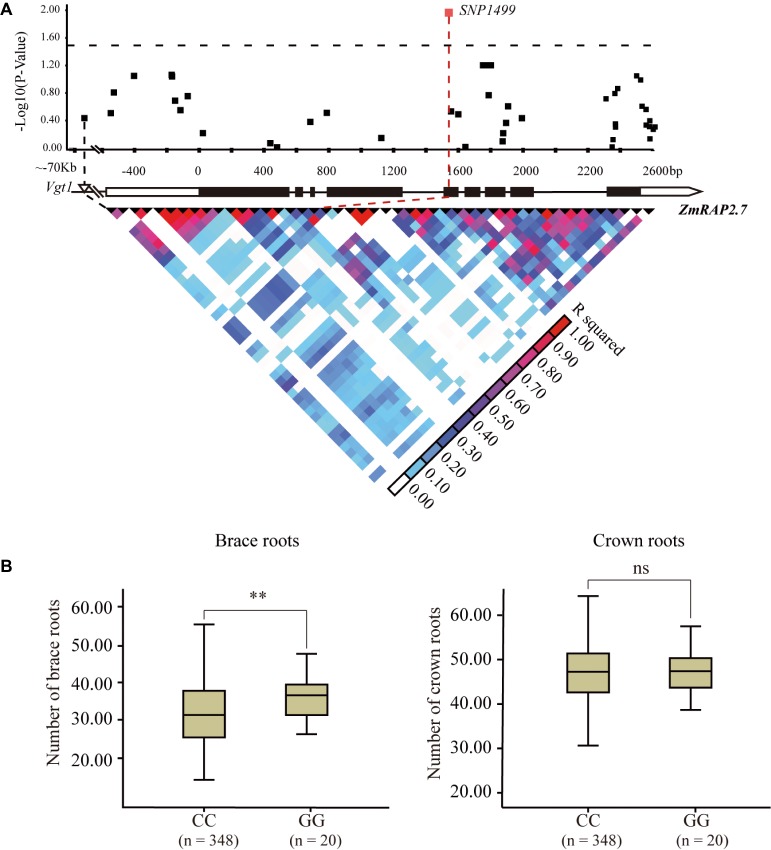
Natural variation in *ZmRAP2.7* gene was significantly associated with number of brace roots within a maize association panel (AM508). **(A)** *ZmRAP2.7*-based association mapping and pairwise linkage disequilibrium (LD) analysis. The dots represent SNPs. A SNP variant at fifth exon (*SNP1499*) associated with number of brace roots was highlighted in red. **(B)** Number of crown roots and brace roots in haplotypes of *ZmRAP2.7* (GG and CC alleles at *SNP1499*). *n* denotes the number of genotypes belonging to each haplotype group. Significant difference within each group was indicated by an asterisk (^**^p < 0.01) according to Student’s *t*-tests, and no significant was indicated as *n.s.*

The candidate gene association analysis for *ZmRAP2.7* was then conducted in a maize panel (AM508) consisted of 508 diverse lines (Figure 7). The allele variation of *ZmRAP2.7* among these genotypes was extracted according to the corresponding SNPs from the database^8^ (Yang et al., 2011). A total of 49 SNPs, spanning 5′- and 3′-untranslation region (UTR) and 9 exons of *ZmRAP2.7* were identified among these lines. Using the mixed linear model, the *SNP1499*, which located at the fifth exon of *ZmRAP2.7* gene, revealed a significant association with number of brace roots (Figure 7). This *SNP1499* could contribute to 12.5% of the phenotypic variation within the population. By contrast, *SNP1499* was not associated with number of crown roots, in agreement with the role of *ZmRAP2.7* in brace roots development as revealed by mutant analysis. Additionally, the favorite alleles variation identified in *ZmRAP2.7* could contribute to generate molecular marker for selecting the traits for brace roots.

### ZmRAP2.7 Localized in the Nucleus and Revealed Transcription Activity

The *ZmRAP2.7* gene contained an open reading frame of 1,413 bp and encodes a predicted 470 amino acids protein with a predicted molecular weight of 51.66 kDa. ZmRAP2.7 protein was predicted to contain two α-helices (Supplementary Figures S6A,B) with the two conserved AP2/ERF domains (153–215 aa and 245–308 aa) (Supplementary Figure S6C). Thus, the ZmRAP2.7 protein was predicted as one of the members in AP2 transcription factor family.

**Figure 8:**
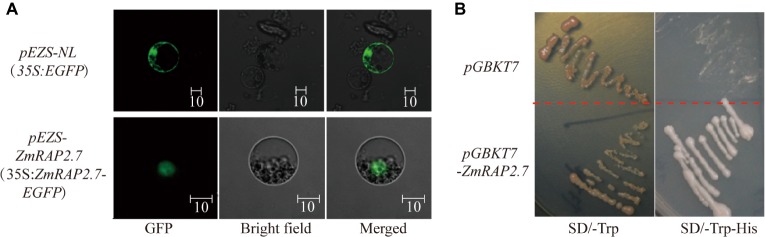
ZmRAP2.7 localized in the nuclear with transcription activity. **(A)** Nuclear localization of ZmRAP2.7 protein in maize. The constructs *pEZS-35S:EGFP* and *pEZS-35S:ZmRAP2.7-EGFP* were transiently expressed into maize mesophyll protoplasts. ZmRAP2.7-dependent GFP fluorescence was observed in the nuclear. The scale bar represents 10 μm. **(B)** Transcription activation of ZmRAP2.7 in yeasts. The constructs *pGKBT7* and *pGKBT7-ZmRAP2.7* were transformed in yeasts and grew for 3 days on the selected medium as indicated.

To investigate the subcellular localization of ZmRAP2.7 protein, the full-length ORFs of *ZmRAP2.7* was fused to EGFP and transiently expressed in maize leaf protoplasts (Figure 8A). In contrast to GFP fluorescence throughout the whole cell, the ZmRAP2.7-dependent green fluorescence was mainly localized in the nucleus (Figure 8A), indicating a nuclear localization of ZmRAP2.7 protein. The yeast strains transformed with the *pGBKT7-ZmRAP2.7* were able to grow on the selected medium SD/-Trp/-His in which those stains with empty vector *pGBDKT7* could not grow (Figure 8B). This result indicated that ZmRAP2.7 revealed the transcriptional activity, suggesting a role of ZmRAP2.7 as a transcription activator.

## Discussion

The brace roots are important component for the whole root system of maize. By growing into the soils, the brace roots contribute significantly to lodging tolerance and water and nutrient uptake efficiency. However, few genes have been cloned for maize brace roots development, and the underlying mechanism remains to be elucidated. In this study, we characterized a maize AP2 transcription factor gene, *ZmRAP2.7*, with an essential function in the brace roots development. In addition, the identified allele variation of *ZmRAP2.7* could be used to generate valuable markers for genetic improvement of root traits.

Several evidences indicated that *ZmRAP2.7* gene was involved in brace roots development in maize. First, *ZmRAP2.7* was mainly expressed in roots (Supplementary Figure S3) and the nodes with initiated brace roots (Figure 2A). Second, the alteration of *ZmRAP2.7* expression resulted in the changes of number of braces roots (Figures 1, 2, 4–6). In contrast to wild type, *RAP2.7-Mu* plants defective in *ZmRAP2.7* expression showed lower number of brace roots (Figures 4–6), while *Corngrass1* plants with the elevated *ZmRAP2.7* expression showed higher number of brace roots (Figures 1A, 2B). Third, ZmRAP2.7 protein was localized in the nuclear and revealed transcriptional activity. However, the downstream genes of *ZmRAP2.7* and how they regulate brace roots development remain largely unknown. As maize *ZmRTCS* and *ZmRTCL* and their upstream regulator *ZmARF35* control shoot-borne roots development (Taramino et al., 2007; Xu et al., 2015), it would be worthy to investigate whether this pathway might be regulated by *ZmRAP2.7*. In addition, two rice AP2 transcription factor genes (*OsERF3* and *OsCRL5*) were found to control shoot-borne crown root initiation and elongation by regulating the response regulators (RRs) of cytokinin signaling (Zhao et al., 2009, 2015; Kitomi et al., 2011). Thus, *ZmRAP2.7* may also regulate cytokinin pathway for controlling shoot-borne roots development in maize.

Many previous studies in maize demonstrated that *ZmRAP2.7* gene was a negative regulator for the flowering time as revealed by genome-wide association analysis (Buckler et al., 2009), QTL analysis (Castelletti et al., 2014) and transgenic analysis (Salvi et al., 2007). The transgenic maize lines overexpressing *ZmRAP2.7* revealed a delayed flowering time and also an increase of node number (Salvi et al., 2007). Meanwhile, the SNPs located in the genomic region of *ZmRAP2.7* were found to be significantly associated with the node number within a nested association mapping (NAM) population (Wallace et al., 2014). Thus, a role of *ZmRAP2.7* in regulating the node number could be supposed besides the flowering time. The alternations of *ZmRAP2.7* expression in *Corngrass1* or *RAP2.7-Mu* plants resulted in changes of the number of brace roots associated with the node numbers (Figures 1, 5, 6). Because *miR156* and *miR172* pathways control the transition between juvenile and adult stage (Lauter et al., 2005; Chuck et al., 2007b; Yang et al., 2011; Navarro et al., 2017), the downstream gene *ZmRAP2.7* might function in keeping the stem nodes at juvenile stage to maintain their meristematic ability for developing brace roots.

As *ZmRAP2.7* was involved in controls of both brace roots development and flowering time, a pleiotropy effect of *ZmRAP2.7* could be expected. Using a maize association panel (AM508), number of brace roots was found to be significantly associated with several developmental traits including the date for heading, silking, and pollen shed (Supplementary Table S2; Li et al., 2013; Yang et al., 2014). This phenotypic correlation suggested a genetic relationship between brace roots and flowers development in maize, which could be explained by the pleiotropy function of *ZmRAP2.7* in both developmental processes. Indeed, *RAP2.7-Mu* plants showed an early flowering time and less number of brace roots (Figures 5, 6; Supplementary Table S3). QTL analysis of a teosinte-maize population also established a genetic association between number of shoot-borne roots and flowering time and identified *ZmCCT* gene co-regulating both traits (Zhang et al., 2018). A similar case was also found in wheat and barley that loss function of *VERNALIZATION1* (*VRN1*) leads to promote flowering time and root phenotypes (Deng et al., 2015; Voss-Fels et al., 2017).

However, the functions of *ZmRAP2.7* in brace roots and flower development might be regulated independently. Within AM508, the *SNP1499* was significantly associated with number of brace roots, but not associated with the date of heading, silking, and pollen shed (Supplementary Figure S7). Previous studies showed that *Vegetative to generative transition 1* (*Vgt1*), a 2-Kb noncoding region positioned 70 Kb upstream of *ZmRAP2.7*, could function as a cis-acting regulatory element to repress the *ZmRAP2.7* expression level and resulted in an earlier flowering time (Salvi et al., 2007; Castelletti et al., 2014; Navarro et al., 2017). Indeed, within AM508, the *Vgt1* allelic variation could also contribute to the phenotypic variation of flowering time. However, *Vgt1* allelic variation was not associated with the number of brace roots (Supplementary Figure S8). Thus, the distinct allelic variation of *ZmRAP2.7* could be supposed to regulate brace roots or flowers development independently, but the underlying mechanism remains to be elucidated.

Taken together, this study characterized a maize AP2 transcription factor, ZmRAP2.7, with essential functions in brace roots development. *ZmRAP2.7* could stimulate brace roots development and also repress the flowering time. In addition, natural variations of *ZmRAP2.7* associated with root traits allow to develop the molecular markers for improving root systems in maize.

## Data Availability

The datasets generated for this study are available on request to the corresponding author.

## Author Contributions

JL and LY conceived and designed the experiments. JL conducted most of the experiments. FC performed the field trials. YL and YW genotyped and phenotyped the *RAP2.7-Mu* mutant. PL collected the root phenotypes in AM508 maize panel. JL and LY analyzed the data and wrote the manuscript. GM helped to revise the manuscript. All authors read and proved the manuscript.

### Conflict of Interest Statement

The authors declare that the research was conducted in the absence of any commercial or financial relationships that could be construed as a potential conflict of interest.

## Footnotes

^1^http://www.maizegdb.org/documentation/uniformmu/

^2^https://phytozome.jgi.doe.gov/

^3^http://smart.embl-heidelberg.de/

^4^http://bioinf.cs.ucl.ac.uk/psipred/

^5^http://swissmodel.expasy.org/

^6^planttfdb.cbi.pku.edu.cn

^7^http://www.maizego.org/

^8^http://www.maizego.org/Resources.html

## Abbreviations

AT: Allotetraploidization
BR: Brace roots
*CG1*: *Corngrass1*
CR: Crown roots
*CRL*5: *CROWNROOTLESS5*
LD: Linkage disequilibrium
QTL: Quantitative trait locus
RR: Response regulators of cytokinin signaling
RSA: Root system architecture
*rtcs*: *Rootless concerning crown and seminal roots*
SD: Segments duplicated
SNP: Single-nucleotide polymorphism
*Tp1*: *Teopod1*
*Tp2*: *Teopod2*
UTR: Untranslated region
*Vgt1*: *Vegetative to generative transition 1*
*VRN1*: *VERNALIZATION1.*
