# Supplementary material for: ZmRAP2.7, an AP2 Transcription Factor, Is Involved in Maize Brace Roots Development
Source: Front Plant Sci. 2019 Jul 4;10:820. doi: 10.3389/fpls.2019.00820 (PMC6621205; doi:10.3389/fpls.2019.00820)
Supplement: Supplementary file 1 [file Data_Sheet_1.PDF]

ZmRAP2.7, an AP2 transcription factor, is involved in maize brace roots development

Jieping Li[1, 2], Fanjun Chen[1], Yanqing Li[1], Pengcheng Li[1, 3], Yuanqing Wang[1], Guohua Mi[1], Lixing Yuan[1,*]

[1] Key Lab of Plant-Soil Interaction, MOE, College Resources and Environmental Sciences, China Agricultural University, Beijing, China, 100193

[2] Key Laboratory of Plant Stress Biology, State Key Laboratory of Cotton Biology, School of Life Sciences, Henan University, Kaifeng, Henan Province, China, 475001

[3] Jiangsu Key Laboratory of Crop Genetics and Physiology/Co－Innovation Center for Modern Production Technology of Grain Crops, Key Lab of Plant Functional Genomics, MOE, Yangzhou University, Yangzhou, China, 225009

* To whom correspondence should be addressed. E-mail: yuanlixing@cau.edu.cn; Tel: 86-010-62734424; Fax: 86-10-62731016;

Supplementary data:

Supplementary Figure S1-8

Supplementary Table S1-3

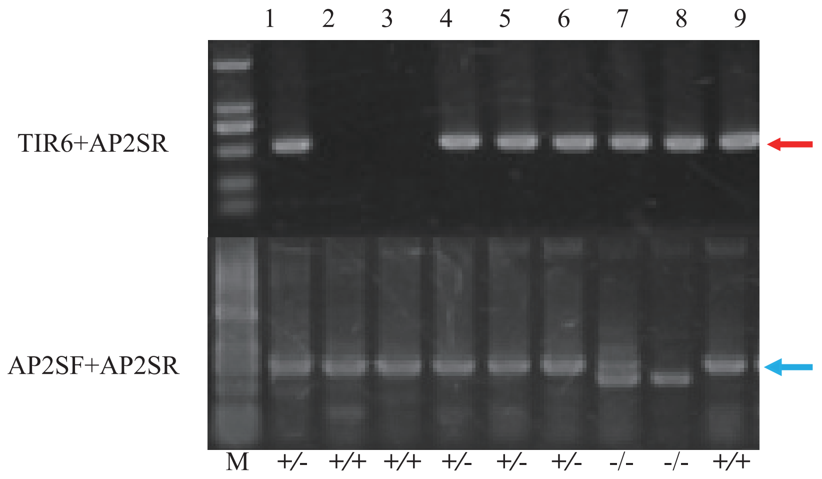

Supplementary Figure S1. Genotypic characterization of *RAP2.7-Mu* transposon insertion mutant. Each genotype was analyzed using the primers sets AP2SR+TIR6 and AP2SF +AP2SR for amplifying Mu transposon insertion-specific band (with size of ~500 bp signed by red arrow) and gene-specific band (with size of ~750 bp signed by blue arrow), respectively. The homozygous wild type (+/+, Lane 2-3) revealed the Mu-transposon-related band, the homozygous mutant *RAP2.7-Mu* (-/-, Lane 7-8) with the gene-related band, and the heterozygote genotypes (+/-, Lane 1 and 4-6) with both bands.

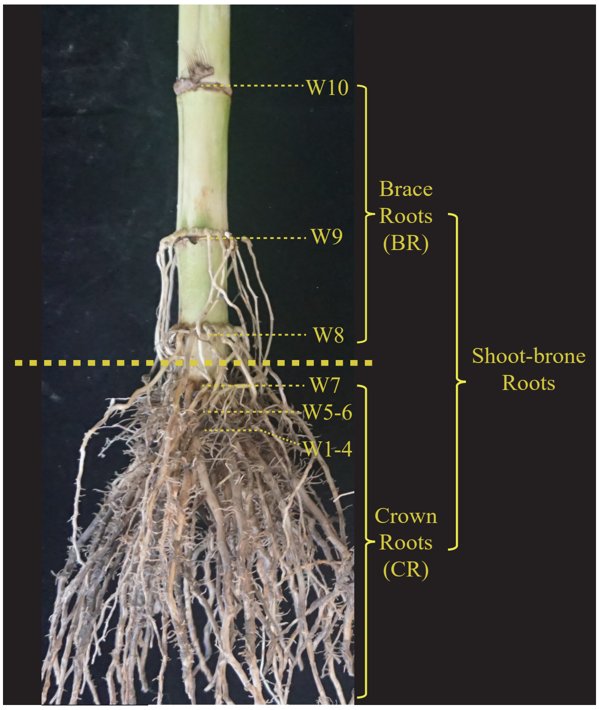

Supplementary Figure S2. Root system architecture of field-grown maize plants at mature stage.
Maize plants (line W22) were grown in the field for 75 days after sowing, and the whole root systems were extracted from soils. Shoot-borne roots are initiated at the consecutive shoot nodes, and consist of crown roots from belowground nodes [whorl (W) 1-4] and brace roots from aboveground nodes [whorl (W) 5-7].

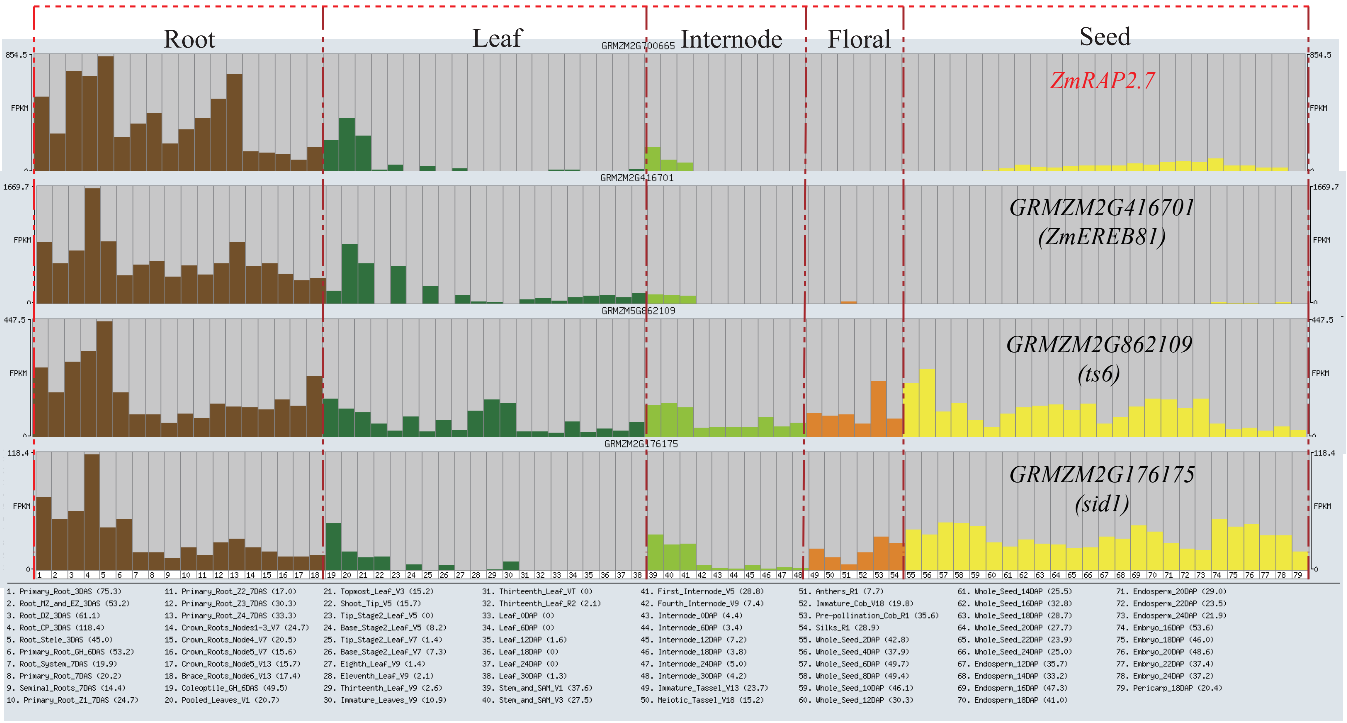

Supplementary Figure S3. Expression pattern of maize *ZmRAP2.7* gene and other three homologous genes within the family.

The data were obtained from the database (http://www.maizegdb.org/; Stelpflug *et al*., 2016). Two genes, *GRMZM2G862109* (*ts6*) and *GRMZM2G176175* (*sid1*), were constitutively expressed in the most of organs included root, leaf, internode, floral and seed. By contrast, the genes *GRMZM2M2G700665* (*ZmRAP2.7*) and *GRMZM2G416701* (*ZmEREB81*) were abundant in roots.

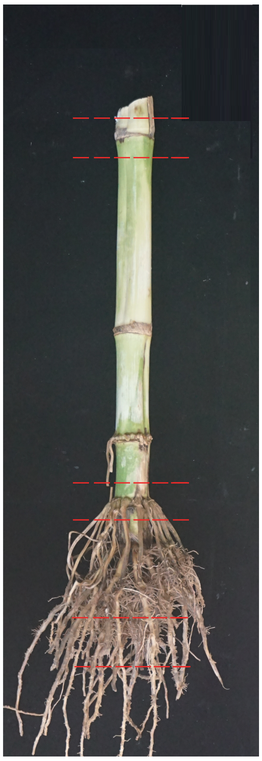

Supplementary Figure S4. Tissue samples collected for *ZmRAP2.7* gene expression analysis in Figure 2. Maize plants for each genotype were grown in the field for 70 days after sowing, and the whole root systems were extracted from soils. Three types of tissues, included root (R), node with brace roots (NBR), and node without brace roots (N), were sampled.

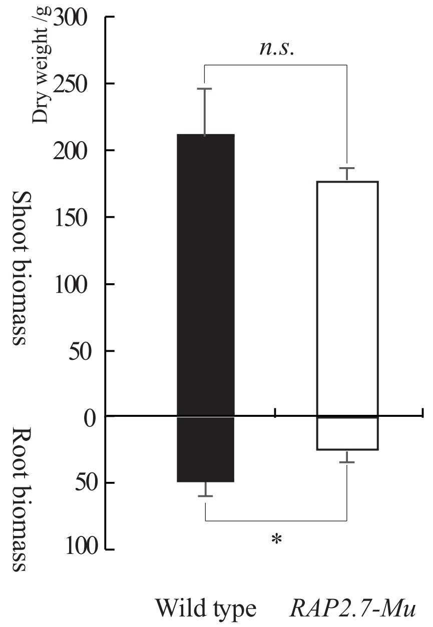

Supplementary Figure S5. Decreased root biomass, but not shoot biomass, in *RAP2.7-Mu* mutant. Root and shoot biomass of *RAP2.7-Mu* mutant and wild type W22 plants grown in Shangzhuang (2016) at mature stage. Bars indicate mean ± SD (n=9). Significant difference was indicated by an asterisk (*, $P<0.05$) according to Student's t-tests, and no significance was indicated by *n.s.*.

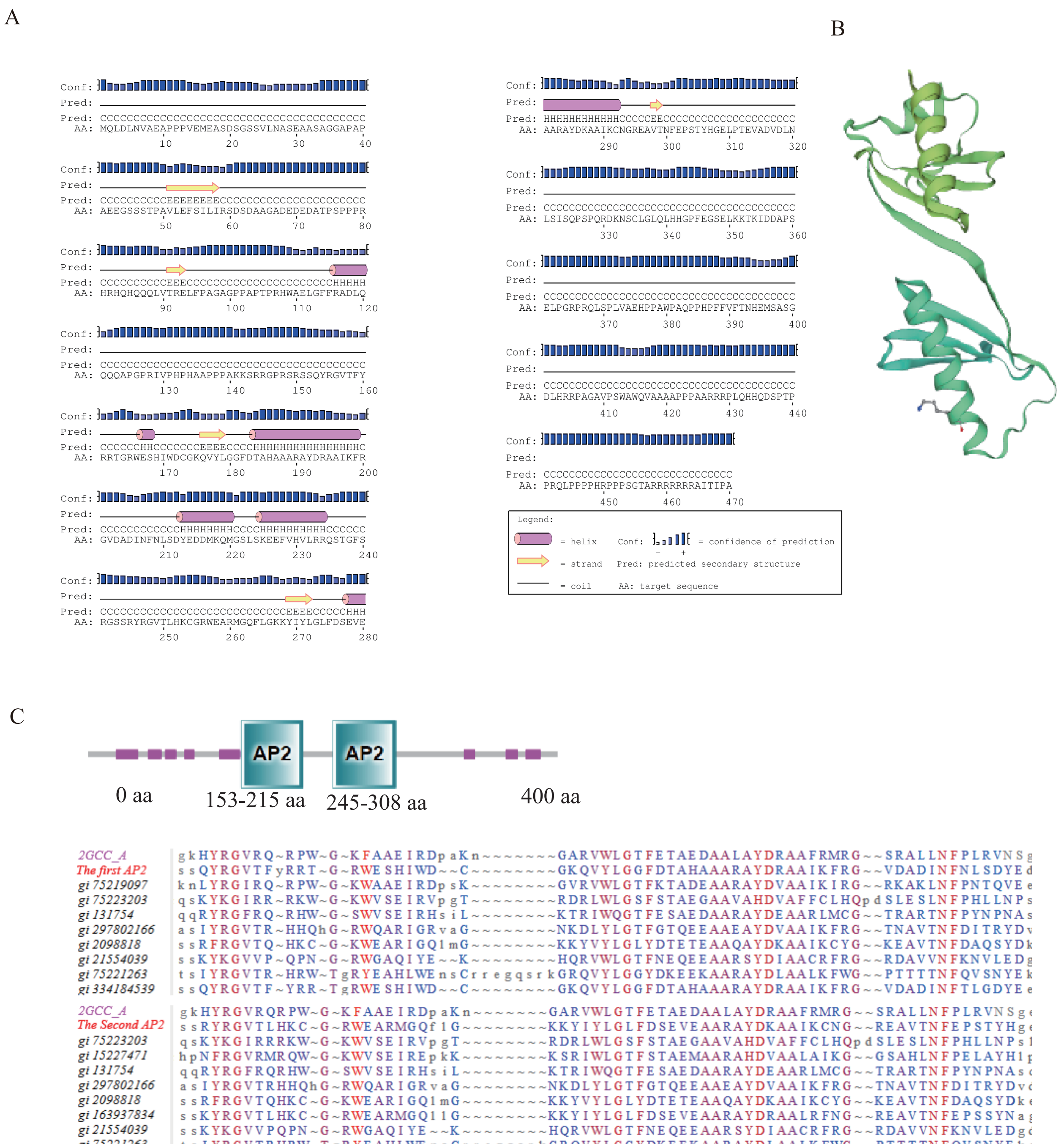

Supplementary Figure S6. Predicted structure of ZmRAP2.7 protein
(A) Secondary structure and (B) tertiary structure of ZmRAP2.7 protein. (C) Two AP2 domains (153-215 aa and 245-308 aa) and the conserved sequence in two AP2 domains.

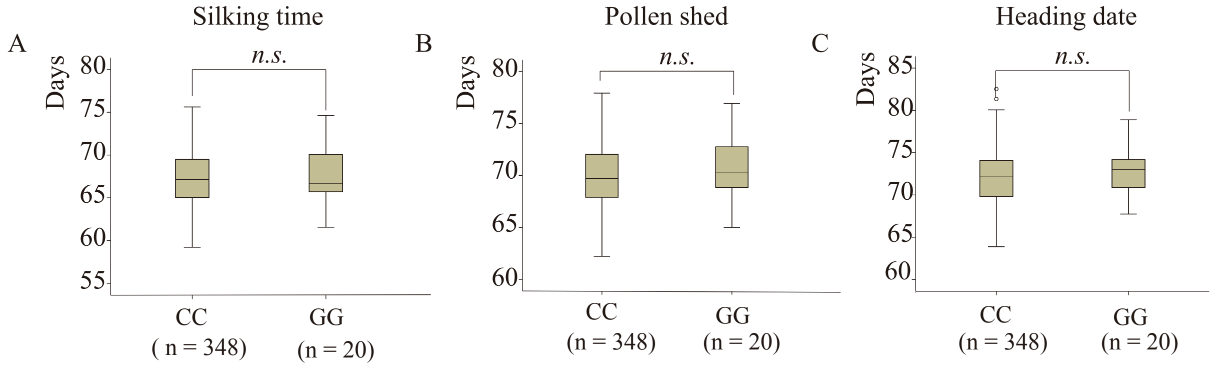

Supplementary Figure S7. Flowering traits between two haplotypes of *ZmRAP2.7* at the *SNP1499* associated with brace roots number within a maize association panel (AM508).
Days of silking (A) , pollen shed (B) and heading date (C) between two haplotypes of *ZmRAP2.7* (GG and CC alleles at *SNP1499*). n denotes the number of genotypes belonging to each haplotype group. No significant difference (*n.s.*) within each group was observed according to Student's t-tests.

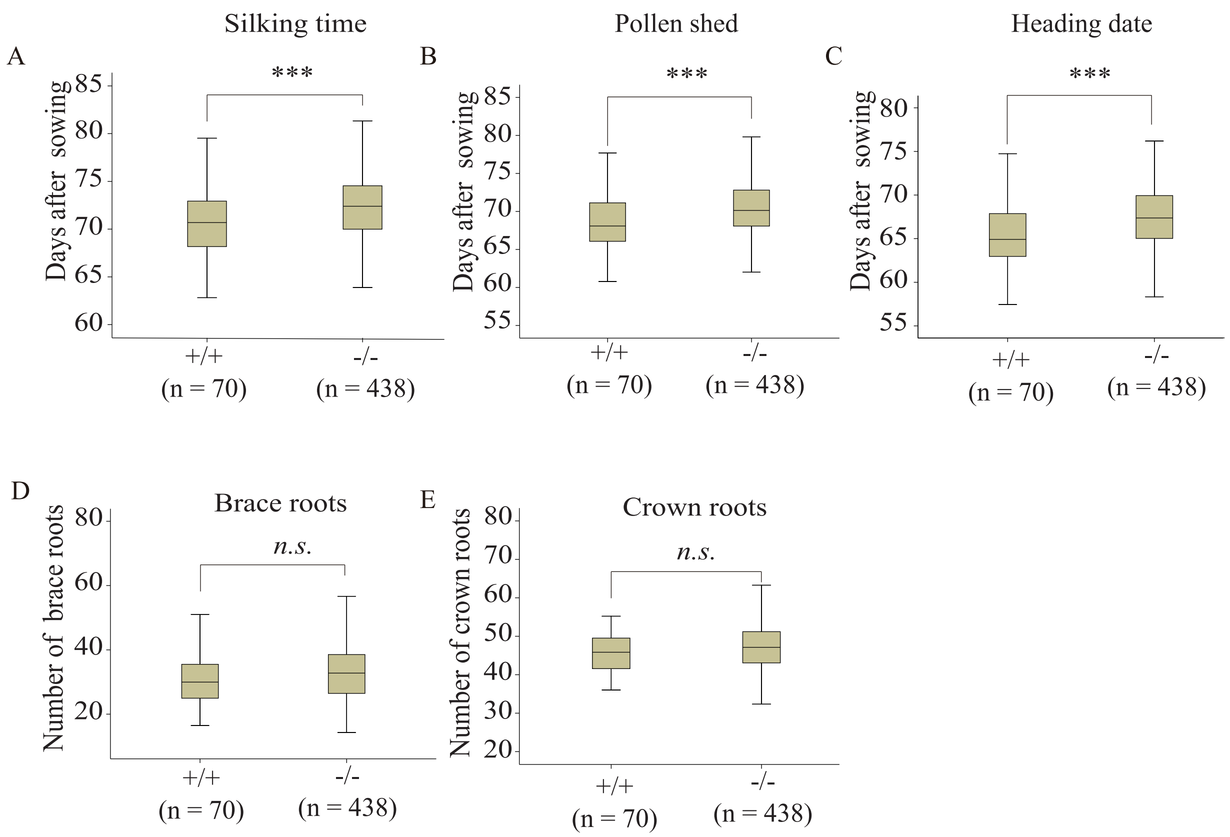

Supplementary Figure S8. Flowering and root traits between two *Vgt1* alleles associated with flowering time within a maize association panel (AM508).

Days of silking time (A) , pollen shed time (B) , heading date (C) , number of brace roots (D) , and number of crown roots (E) between two *Vgt1* alleles which contained MITE transposon (+/+) or not (-/-). Significant difference within each group was indicated by an asterisk (***, P<0.001) according to Student's t-tests, and no significant was indicated as *n.s.*.

Supplementary Table S1. List of primers used in this study.

**Gene**	**Purpose**	**Accession number**	**Primer pair（5'-3')**
ZmRAP2.7	Full length ORF	*GRMZM2G700665*	ZmRAP2.7CDSF: TAACCGGTCGTCCTCTTCAC
			ZmRAP2.7CDSR: GGAAAATGCTAGCTTCCCGG
ZmRAP2.7	qPCR	*GRMZM2G700665*	ZmRAP2.7RTF: GTCTTCACAAACCATGAGATGAG
			ZmRAP2.7RTR: TGGAGAATCCTGATGATGCTG
ZmRAP2.7	Clone vector	*GRMZM2G700665*	RAP2.7ECR: CGGAATTCATGCAGTTGGATCTGAACGTGG
			RAP2.7BAR: CGGGATCCCGGCGGGGATGGTGATGG
ZmRAP2.7	Genotype	*GRMZM2G700665*	AP2SF: CGTCACCTTCTACCGCCG
			AP2SR: GACGCCTCTGTACCTGGAG
ZmTUB	qPCR	*AJ420856.1*	ZmTUBF: GCTATCCTGTGATCTGCCCTGA
			ZmTUBR: CGCCAAACTTAATAACCCAGTA
TIR	Genotype	*TIR*	TIR6: AGAGAAGCCAACGCCAWCGCCTCYATTTCGTC

Supplementary Table S2. Phenotypic correlation analysis of shoot-borne root number (SBRN)，brace roots number (BRN) and crown roots number (CRN) with other agronomic traits within a maize association panel (AM508).

**Traits**		**PH**	**EH**	**EW**	**EL**	**TML**	**TBN**	**LNA**	**EL**	**EW**	**CD**	**KNR**	**GW**	**CW**	**KW**	**ST**	**PT**	**HD**	**SBRN**	**BRN**	**CRN**
SBRN	Pearson	.162	.237	.130	.286	.056	.212	.197	.028	-.024	.032	-.023	.020	.025	.070	.244	.292	.275	1	.886	.631
	p-Value	.000	.000	.004	.000	.221	.000	.000	.535	.604	.480	.618	.655	.582	.125	.000	.000	.000		.000	.000
BRN	Pearson	.199	.266	.147	.303	.084	.228	.171	.055	-.050	.010	-.011	.025	.034	.026	.223	.263	.246	.886	1	.296
	p-Value	.000	.000	.001	.000	.067	.000	.000	.226	.278	.833	.802	.588	.461	.568	.000	.000	.000	.000		.000
CRN	Pearson	-.011	.026	.050	.107	-.005	.083	.166	-.014	.004	.029	-.006	-.005	-.004	.083	.147	.187	.179	.631	.296	1
	p-Value	.807	.572	.275	.019	.918	.069	.000	.759	.935	.527	.889	.906	.936	.070	.001	.000	.000	.000	.000	

Morphological traits: PH, plant height; EH, ear height; EW, Ear leaf width; EL, Ear leaf length; ED, Ear diameter; TML, Tassel main axis length; TBN, Tassel branch number; LNA, Leaf number above ear; Yield traits: EL, Ear length; ED, Ear diameter; CD, Cob diameter; KNR, Kernel number per row; GW, 100 grain weight; CW, Cob weight; KW, Kernel width;

Developmental traits: ST, Silking time; PT, Pollen shed; HD, Heading date;

Root traits: SBRN, Shoot-borne roots number, BRN, Brace roots number; CRN, Crown roots number;

Pearson: Pearson correlation. *p*-Value, Significant correlation was labelled in red.

Supplementary Table S3. Pollen shed time (days after sowing, das) between wild type and *RAP2.7-Mu* .

**Field trials**	**Wild type**	***RAP2.7 -Mu***
Shangzhuang, 2016	67.3	59.2
Shangzhuang, 2017	69.4	65.2
